# Midkine as a Biomarker in Cardiovascular-Surgery: from old Links to the Newer Insights!

**DOI:** 10.21470/1678-9741-2020-0446

**Published:** 2021

**Authors:** Jasvinder Kaur Kohli, Rohan Magoon, Iti Shri, Ramesh Kashav

**Affiliations:** 1 Department of Cardiac Anaesthesia, Atal Bihari Vajpayee Institute of Medical Sciences (ABVIMS) and Dr. Ram Manohar Lohia Hospital, Baba Kharak Singh Marg, New Delhi, India.; 2 Department of Cardiac Anaesthesia, Atal Bihari Vajpayee Institute of Medical Sciences (ABVIMS) and Dr. Ram Manohar Lohia Hospital, Baba Kharak Singh Marg, New Delhi, India. E-mail: rohanmagoon21@gmail.com; 3 Department of Cardiac Anaesthesia, Atal Bihari Vajpayee Institute of Medical Sciences (ABVIMS) and Dr. Ram Manohar Lohia Hospital, Baba Kharak Singh Marg, New Delhi, India.; 4 Department of Cardiac Anaesthesia, Atal Bihari Vajpayee Institute of Medical Sciences (ABVIMS) and Dr. Ram Manohar Lohia Hospital, Baba Kharak Singh Marg, New Delhi, India.

*To The Editor,*

*Heparin continues to behold a pivotal position in the safe conduct of cardiovascular surgery, but how about the role of heparin-releasable proteins?*

The story dates back to 1943, when Hahn elucidated the abolition of alimentary lipemia following heparin injection in dogs ^[1]^. While this lipid clearance was attributed to the activity of lipoprotein lipase, a range of proteins have been discovered to increase in plasma after intravenous heparin administration, ever since. The family of these proteins falls under the common category of ‘heparin-releasable proteins’(HRPs). Novotny et al. identified novel HRPs in their chromatography-based study in 1993, including midkine, a cytokine-related growth factor with pleiotropic effects (neurotrophic,angiogenesis-stimulating properties, mitogenic, etc.) and peculiarly preserved across the species ^[2]^. Although first cloned in 1988 by Kadomatsu et al, the first description of natural midkine as an HRP captivated attention. ^[2-4]^

The ongoing search for the HRP physiological significance unveiled that these proteins display the ability to bind glycosaminoglycans like heparin to the endothelial cell surfaces. The aforementioned prompted the theory that these HRPs like midkine are intricately linked to the functionality of the endothelium-blood interface ^[2]^. Being a soluble growth factor, detected in healthy individuals in a range of 0-625 pg/ml in serum or plasma, midkine over-expression has been associated with a range of malignancies, septic shock, acute kidney injury (AKI) and diverse ischemic/inflammatory and auto-immune diseases ^[4]^.

Interestingly, the spectrum of midkine elevation presents a colossal opportunity to employ it as a biomarker in a predisposed setting such as cardiovascular surgery (in addition to the extensive research on its role in oncological settings) ^[4]^. In this context, an initial encouraging literature is accumulating with regard to postoperative AKI associated with cardiovascular interventions (particularly multi-factorial in aetiology) ^[5-9]^.Hayashi et al. ^[6]^ revealed that a urinary midkine>11.5 pg/ml outperformed the AKI predictive value of three other biomarkers (area under the receiver operating characteristic [AU-ROC]: midkine 0.88; neutrophil gelatinase-associated lipocalin [NGAL]0.84; interleukin-18 [IL-18]0.72 and N-acetyl-D glucosaminidase [NAG]0.70) in a cohort of 549 patients with AKI. As an extension, the research group also prospectively investigated the utility of midkine for early prediction of AKI in a subset of forty patients undergoing abdominal aortic aneurysm repair. With the urinary biomarker assessment performed at baseline, during aortic cross-clamping and de-clamping, at the end of the procedure and on the 1^st^ post-procedural day, the midkine was characterized by an early pattern of increase compared to the rest of biomarkers ^[6]^.

The importance of urinary biomarkers such as midkine is further emphasized by the novel recognition of the prognostic implications of a single-biomarker positive and risk, injury,failure, loss, end-stage renal disease (RIFLE) criteria negative (subclinical AKI phenotype) in cardiac surgical setting. In a German multi-center study by Albert et al. ^[7]^, midkine positivity detected 16.9% cases with subclinical AKI who demonstrated an elevated risk of eventual renal replacement therapy or in-hospital mortality.Urinary biomarkers have also been found to complement the Cleveland score for adverse kidney event prediction after cardiac surgery ^[8]^. Moreover, McBride et al. ^[9]^have recently highlighted the significance of combining the biomarker risk score with the clinical risk score for peri-operative AKI risk stratification in their prospective evaluation of 401 patients scheduled for cardiac surgery. A combination of post-operative midkine, heart-type fatty-acid binding protein (H-FABP) and soluble tumour necrosis factor receptor 2 (sTNFR2) demonstrated the highest AKI predictive value in their study (AU-ROC:0.836) ^[9]^.

The fact that midkine can be readily quantified in blood or body fluids like urine and cerebrospinal fluid bestows the former with a potential of evolving as a convenient, non-invasive biomarker for the diagnosis, monitoring, prevention and management of the peri-operative cardiovascular complications ^[4]^.However, there are peculiar pre-requisites, like the requirement of sufficient high-quality evidence emanating from multiple larger studies. At the same time, research in the area of midkine’s ability to predict the consequences of ischemia-reperfusion injury in cardiovascular surgeries merits extra attention. Lastly, endeavours like formulating and validating urinary-midkine AKI predictive cut-offs constitute a major step in the direction of an augmented test specificity, particularly when the blood levels can be elevated owing to a range of diseases.

Nevertheless, with novel platforms endorsing the feasibility of simultaneous assessment of a combination of biomarkers in a single clinical sample (‘multiplexing’) ^[4]^, there is a substantial scope for the future development of midkine-based predictive models aimed at the betterment of high-risk cardiovascular surgical subset.

## References

[1] Hahn PF (1943). Abolishment of alimentary lipemia following injection of heparin. Science.

[2] Novotny WF, Maffi T, Mehta RL, Milner PG (1993). Identification of novel heparin-releasable proteins, as well as the cytokines midkine and pleiotrophin, in human postheparin plasma. Arterioscler Thromb.

[3] Kadomatsu K, Kishida S, Tsubota S (2013). The heparin-binding growth factor midkine the biological activities and candidate receptors. J Biochem.

[4] Jones DR (2014). Measuring midkine the utility of midkine as a biomarker in cancer and other diseases. Br J Pharmacol.

[5] Magoon R, Dey S, Walian A, Kashav R (2020). Nitric oxide renoprotective in cardiac surgery!. Braz J Cardiovasc Surg.

[6] Hayashi H, Sato W, Kosugi T, Nishimura K, Sugiyama D, Asano N (2017). Efficacy of urinary midkine as a biomarker in patients with acute kidney injury. Clin Exp Nephrol.

[7] Albert C, Albert A, Kube J, Bellomo R, Wettersten N, Kuppe H (2018). Urinary biomarkers may provide prognostic information for subclinical acute kidney injury after cardiac surgery. J Thorac Cardiovasc Surg.

[8] Albert C, Albert A, Kube J, Bellomo R, Wettersten N, Kuppe H (2018). Urinary biomarkers may provide prognostic information for subclinical acute kidney injury after cardiac surgery. J Thorac Cardiovasc Surg.

[9] McBride WT, Kurth MJ, McLean G, Domanska A, Lamont JV, Maguire D (2019). Stratifying risk of acute kidney injury in pre and post cardiac surgery patients using a novel biomarker-based algorithm and clinical risk score. Sci Rep.

